# Initial inflammatory response after the pulpotomy of rat molars with MTA or ferric sulfate

**DOI:** 10.1590/1678-7757-2018-0550

**Published:** 2019-07-29

**Authors:** Camila Soares LOPES, Marina Azevedo JUNQUEIRA, Leopoldo COSME-SILVA, Camila de Oliveira Rodini PEGORARO, Cássia Cilene Dezan GARBELINI, Thais Marchini OLIVEIRA, Natália Silva MARTINS, Juliana dos Santos NEVES, Vivien Thiemy SAKAI

**Affiliations:** 1 Universidade Federal de Alfenas (UNIFAL-MG), Faculdade de Odontologia, Departamento de Clínica e Cirurgia, Alfenas, Minas Gerais, Brasil.; 2 Universidade do Estado de São Paulo (UNESP), Faculdade de Odontologia de Araçatuba, Departamento de Endodontia, Araçatuba, São Paulo, Brasil.; 3 Universidade de São Paulo, Faculdade de Odontologia de Bauru, Departamento de Ciências Biológicas, Bauru, São Paulo, Brasil.; 4 Universidade Estadual de Londrina, Departamento de Medicina Bucal e Odontopediatria, Londrina, Paraná, Brasil.; 5 Universidade de São Paulo, Faculdade de Odontologia de Bauru, Departamento de Odontopediatria, Ortodontia e Saúde Coletiva, Bauru, São Paulo, Brasil.; 6 Universidade Federal de Alfenas (UNIFAL-MG), Instituto de Exatas, Departamento de Estatística, Alfenas, Minas Gerais, Brasil.; 7 Universidade Federal de Alfenas (UNIFAL-MG), Departamento de Biologia Estrutural, Alfenas, Minas Gerais, Brasil.

**Keywords:** Materials testing, Ferric sulfate, Pulpotomy, Interleukin 6

## Abstract

**Purpose:**

To compare, both qualitatively and quantitatively, the inflammatory cells, vascular density and IL-6 immunolabeled cells present in the pulp after pulpotomy with white MTA versus 15.5% ferric sulfate (FS).

**Methodology:**

Forty-eight mandibular first molars from 24 Wistar rats were divided into MTA or FS groups and subdivided according to the period after pulpotomy procedure (24, 48 and 72 hours). Four teeth (sound and untreated) were used as controls. Histological sections were obtained and assessed through the descriptive analysis of morphological aspects of pulp tissue and the quantification of inflammatory cells, vascular density and interleukin-6 (IL-6) expression. Data were statistically analyzed (p<0.05).

**Results:**

The number of inflammatory cells was similar in both groups, being predominantly localized at the cervical radicular third. In the MTA group, increased inflammation was observed at 48 hours. Vascular density was similar in both groups and over time, being predominant in the medium radicular third. No correlation was found between the number of inflammatory cells and the vascular density. Pulp tissue was more organized in MTA-treated teeth. In both groups, a weak to moderate IL-6 expression was detected in odontoblasts and inflammatory cells. Comparing both groups, there was a greater IL-6 expression in the cervical radicular third of teeth treated with MTA at 24 hours and in the medium and apical thirds at 72 hours, while in the FS group a greater IL-6 expression was found in the apical third at 24 hours.

**Conclusion:**

The MTA group presented better histological features and greater IL-6 expression than the FS group. However, no difference was observed between the groups regarding the inflammatory status and vascularization, suggesting the usefulness of FS as a low-cost alternative to MTA.

## Introduction

Pulpotomy consists of the coronal pulp amputation and treatment of remaining radicular tissue with capping agents that preserve its vitality and function. To keep the vitality of pulp tissue and impair pathological alterations, exposed pulp stumps should be covered with a biocompatible material.^1^

The growing interest in biocompatible materials promoted MTA as the capping material of choice for pulpotomies. In contact with periradicular or dental pulp tissues, MTA is able to maintain pulp vitality and to stimulate repair.^2^ This material also enhances the deposition of mineralized tissue in root canals, thus causing radicular stenosis or pulp canal obliteration.^3,4^ Despite the high clinical, radiographic and histological success rates of MTA, both tooth discoloration^5^ and its high cost^6,7^ are remarkable inconveniences. Ferric sulfate (FS) has been considered an appropriate and low-cost alternative capping material for primary teeth pulpotomy.^1^ This is a coagulative and hemostatic agent that forms ferric ion-protein complex on contact with blood, thus reducing the inflammatory response.^8,9^ The FS presents high clinical and radiographic success rates, comparable to that of formocresol.^10-13^ However, studies assessing the histological condition of the remaining pulp are scarce.^4,14,15^

The analysis of pulp response to capping materials is the best way to determine both their action mechanism as the pulp condition after pulpotomy.^16^ Considering the pulp inflammatory events may favor the initial phases of pulp repair and that the exacerbation of inflammation often leads to necrosis of the entire tissue,^17^ it is important to evaluate the acute pulp response after pulpotomy with different capping materials, to determine the most favorable one. Therefore, the purpose of this study was to compare, both qualitatively and quantitatively, the inflammatory cells, vascular density and IL-6 immunolabeled cells present in the pulp 24, 48 and 72 hours after pulpotomy with white MTA *versus* 15.5% FS.

## Methodology

### Experimental design

The Animal Ethics Committee approved the protocol of this study (570/2014). Considering a prior study,^18^ the sample size of 7 teeth *per* group achieved 95% power and 1% significance level. We added 10% to the final sample size to compensate for possible losses, leading to a final sample size of 8 teeth *per* material in each experimental period.

Mandibular first molars from twenty-six young male rats (*Rattus norvegicus*, *albinus*, Wistar), weighing between 270 and 300 g, were used in the study. A total of 52 mandibular first molars were included in the study. Out of these, 48 teeth were randomly assigned into 2 groups (12 rats/group; 24 teeth/group), according to the capping material employed (MTA and 15.5% FS). Each group was further divided into 3 subgroups (8 teeth/group), considering the period after pulpotomy (24, 48 and 72 hours). In two animals, the molars remained sound or with an opening in the pulp chamber (untreated) performed immediately before euthanasia, thus serving as parameters for the histological description. During all the experimental period, animals were kept in polypropylene cages (four *per* cage) with *ad libitum* access to food (Nutrilabor; Guabi, Campinas, SP, Brazil) and water, and maintained on a 12 h light–dark cycle at 23±2°C temperature and 55±10% humidity. Cage bedding was changed at least three times a week.

### Operation procedure

Animals were anesthetized through muscular injection of a cocktail containing 6 mg/kg xylazine (Anasedan - Sespo Indústria e Comércio Ltda, Paulínia, SP, Brazil) and 70 mg/kg ketamine (Cetamin - Rhobibarme Indústria Farmacêutica Ltda, Hortolândia, SP, Brazil) and were immobilized in an operatory table.

Conventional pulpotomy technique was performed. Briefly, a round diamond bur (#1011, Dentsply Indústria e Comércio Ltda, Petropólis, RJ, Brazil) in a high-speed handpiece was employed to open the pulp chamber, and an excavator was used to remove the coronal pulp tissue (#5, SS White, Dental Objects Ltda., Juiz de Fora, MG, Brazil). Copious irrigation with saline solution was performed until bleeding control was achieved.

In the MTA group, MTA (MTA White, Angelus, Londrina, PR, Brazil) was mixed at a 1:1 powder/distilled water ratio and the paste was placed on the pulp stumps. In the FS group, a sterile cotton pellet saturated with 15.5% ferric sulfate (Astringedent - Ultradent Products Inc, South Jordan, UT, USA) and further compressed between gauzes to remove excess solution was placed on the remaining pulp for 15 seconds; the cotton pellet was removed and pulp stumps were covered with a zinc oxide and eugenol cement (Biodinâmica Quím. e Farm. Ltda, Ibiporã, PR, Brazil). In both groups, conventional glass ionomer cement (Vidrion R – SS White Artigos Dentários Ltda, Rio de Janeiro, RJ, Brazil) was used to seal the cavities.

During the recovery period, animals were kept under observation. To minimize postoperative discomfort, animals received intravenous dipyrone (0.03 mg *per* 100 g of body weight) during the first three days. Feeding and drinking pattern and possible changes in behavioral profile were also assessed. After the experimental periods (24, 48 and 72 hours after pulpotomy), animals received muscular injection of anesthesia (6 mg/kg xylazine and 70 mg/kg ketamine) and were euthanized by CO_2_ inhalation.

### Histological preparation

Mandibles were dissected and teeth were removed in blocks, to include surrounding bone. Specimens were fixed in buffered 4% formaldehyde for 24 hours and decalcified in ethylenediamine tetraacetic acid (EDTA) for 30 days. After dehydration and paraffin embedding, longitudinal 5 µm serial sections were obtained. The specimens were prepared for histology and stained with hematoxylin-eosin (HE) for immunohistochemistry.

#### Morphometric analysis of dentin-pulp complex

Histological sections were analyzed with a binocular optical microscope (AxioLab A1 Plus - Carl Zeiss, Jena, Thuringia, German) with 40X lenses and with the imaging software AxioVision Rel 4.8.2 (Carl Zeiss, Jena, Thuringia, German). Between six and nine images were captured from either the mesial or the distal root of each pulpotomized tooth, comprising two or three images from each radicular third (cervical, medium and apical).

The software Image Pro Plus 4.5 (Media Cybernetics, Silver Spring, MD, USA) was used to quantify the inflammatory cells per image. The same software was employed to analyze vascular density. For this purpose, three 49-point intersection grids were superimposed on the images, and points that overlaid the blood vessels were counted. The following formula was used: vascular density=(number of counted intersection points/total number of points in the grid)x100.

Finally, the mean numbers of inflammatory cells and vascular density were calculated *per* radicular third.

#### Analysis of IL-6 immunoreactivity

Sections were deparaffinized in xylene, re-hydrated, rinsed with phosphate buffer saline (PBS), and then incubated in hydrogen peroxidase (30 vol.) for 30 minutes. The sections were incubated with 1:800 dilution of a mouse monoclonal anti-human IL-6 antibody (ab9324, Abcam, Cambridge, UK), for 12 hours at 4°C, followed by incubation with the biotinylated rabbit anti-mouse secondary antibody at room temperature, and treated with streptavidin peroxidase complex for 1 hour (Universal Dako Labeled HRP Streptavidin-Biotin Kit^®^, Dako Laboratories, Carpinteria, CA, USA). Color was developed with a DAB solution (DAB; Sigma-Aldrich Corp., St. Louis, MO, USA). Tissue sections were counter-stained with hematoxylin and sealed with cover slides using an aqua-poly mounting solution (VectaMount AQ; Vector Laboratories, Inc, Burlingame, CA, USA). As controls, sections were stained with an isotype-matched nonspecific immunoglobulin G (Santa Cruz Biotechnology, Santa Cruz, CA, USA).

Sections were captured in a similar manner to the HE-stained ones and analyzed using the ImageJ software (ImageJ v1.43j; National Institutes of Health, Bethesda, MD, USA). The quantification of IL-6 expression was performed using the color deconvolution technique.^19^ This technique leads to the production of three images (DAB, hematoxylin and a complimentary image). In DAB image, the area of interest (pulp connective tissue) was selected and separated of the remnant field using the image brush tool. Small histological artifacts and large blood vessels were not considered in the selection. A histogram of the delimited area was performed and the quantification of DAB color pixel intensity was automatically set. Pixel intensity values for DAB ranged from 0 to 255, wherein 0 means the darkest shade (positive IL-6 immunoexpression) and 255 mean the lightest shade of the color (negative IL-6 immunoexpression).

#### Descriptive analysis

Descriptive analysis of histological findings was conducted both in the sections stained with HE as in those tested for IL-6 immunoreactivity.

#### Statistical analysis

The mean number of inflammatory cells, vascular density and IL-6 imunoexpression were calculated for each radicular third. Data were tested for normality using the Shapiro-Wilk test. Kruskal-Wallis, Dunn and Spearman correlation tests were conducted to data with nonparametric distribution, whereas T, F and Tukey tests were used for parametric data distribution. Statistical analyses were performed using BioEstat (version 5.3) and R statistical software (version 3.4.3), considering P<0.05.

## Results

The sound teeth presented a normal pulp tissue, characterized by a loose connective tissue containing several types of cells and thin-walled blood vessels in the central area, as well as an odontoblast layer in the periphery. No inflammation was observed in these teeth. In untreated teeth, a mild inflammation, with a small number of neutrophils, was detected near the coronal opening, while the remaining tissue exhibited an aspect of normality.

**Figure 1:**
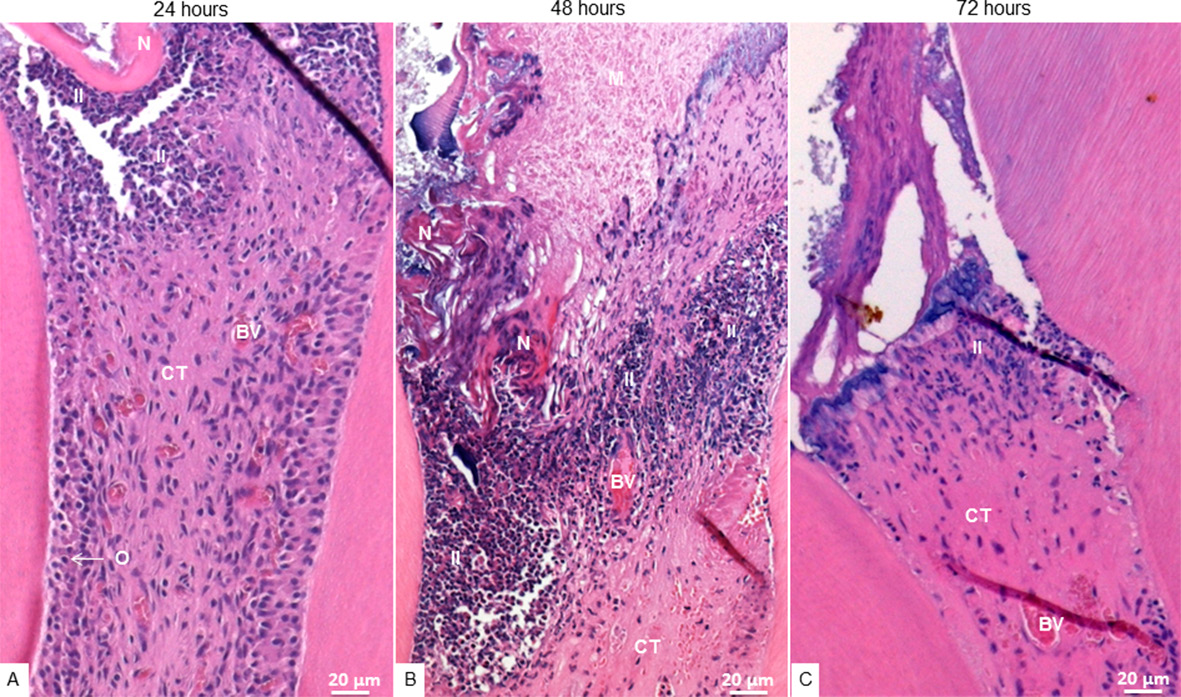
Photomicrographs of hematoxylin and eosin-stained cervical radicular third of MTA-treated teeth. A: At 24 hours, presence of inflammatory infiltrate (II) below the capping material (M), normal connective tissue (CT) with blood vessels (BV) in the central area and odontoblasts (O) in the periphery of the pulp tissue; B: At 48 hours, presence of II predominantly located close to the M and superficial necrosis (N); C: At 72 hours, presence of II in the cervical third and normal CT. 20x magnification

**Figure 2:**
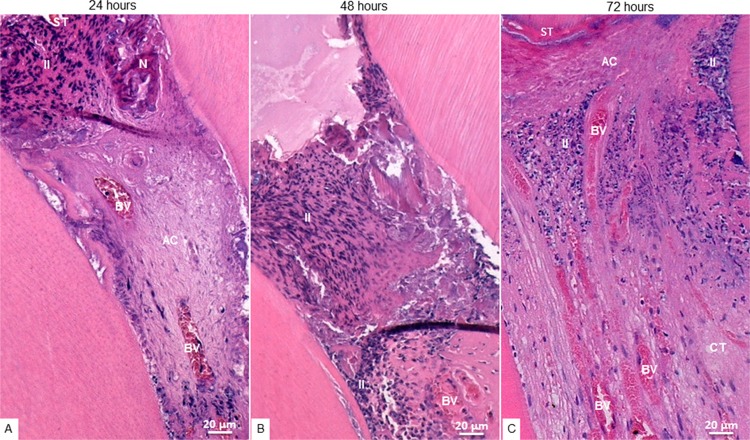
Photomicrographs of hematoxylin and eosin-stained cervical radicular third of SF-treated teeth. A: At 24 hours, presence of superficial tissue (ST) in contact with the material, followed by an inflammatory infiltrate (II), necrosis (N) and an acellular layer (AC); B: At 48 hours, presence of II and disorganized connective tissue (CT); C: At 72 hours, presence of ST in contact with the material, followed by an AC and several congested blood vessels (BV), and dispersing II through the pulp tissue. 20x magnification

In the tested groups and during the three periods of evaluation, the inflammatory response was greater in the cervical third (Figures 1 and 2), gradually decreasing through the medium and apical third. The inflammatory infiltrate, mainly composed of neutrophils, was more intense at 48 hours. Vascularization was predominant in the medium third, with inflammatory cells inside and around the vessels.

**Figure 3:**
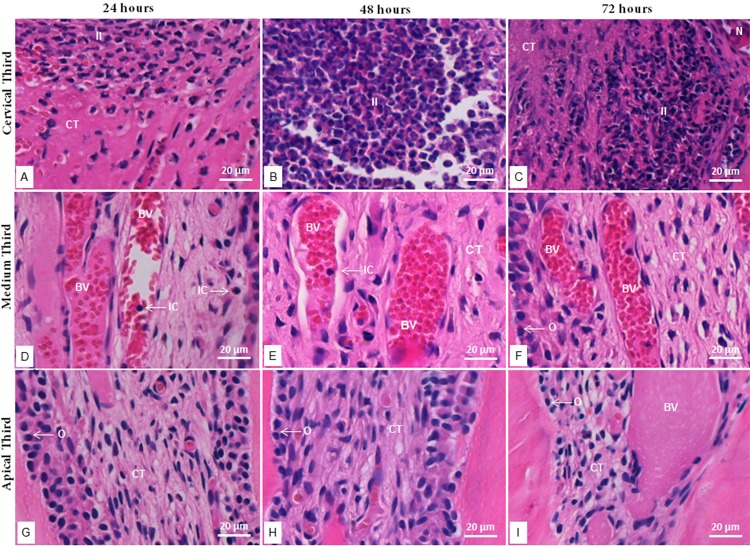
Photomicrographs of hematoxylin and eosin-stained radicular third sections of MTA-treated teeth at 24, 48 and 72 hours. a-c: Cervical radicular third at 24, 48 and 72 hours, respectively; d-f: Medium radicular third at 24, 48 and 72 hours, respectively; g-i: Apical radicular third at 24, 48 and 72 hours, respectively. (II) inflammatory infiltrate; (N) necrosis; (BV) blood vessels; (CT) connective tissue; (O) odontoblast; (IC) inflammatory cell. 40x magnification

**Figure 4:**
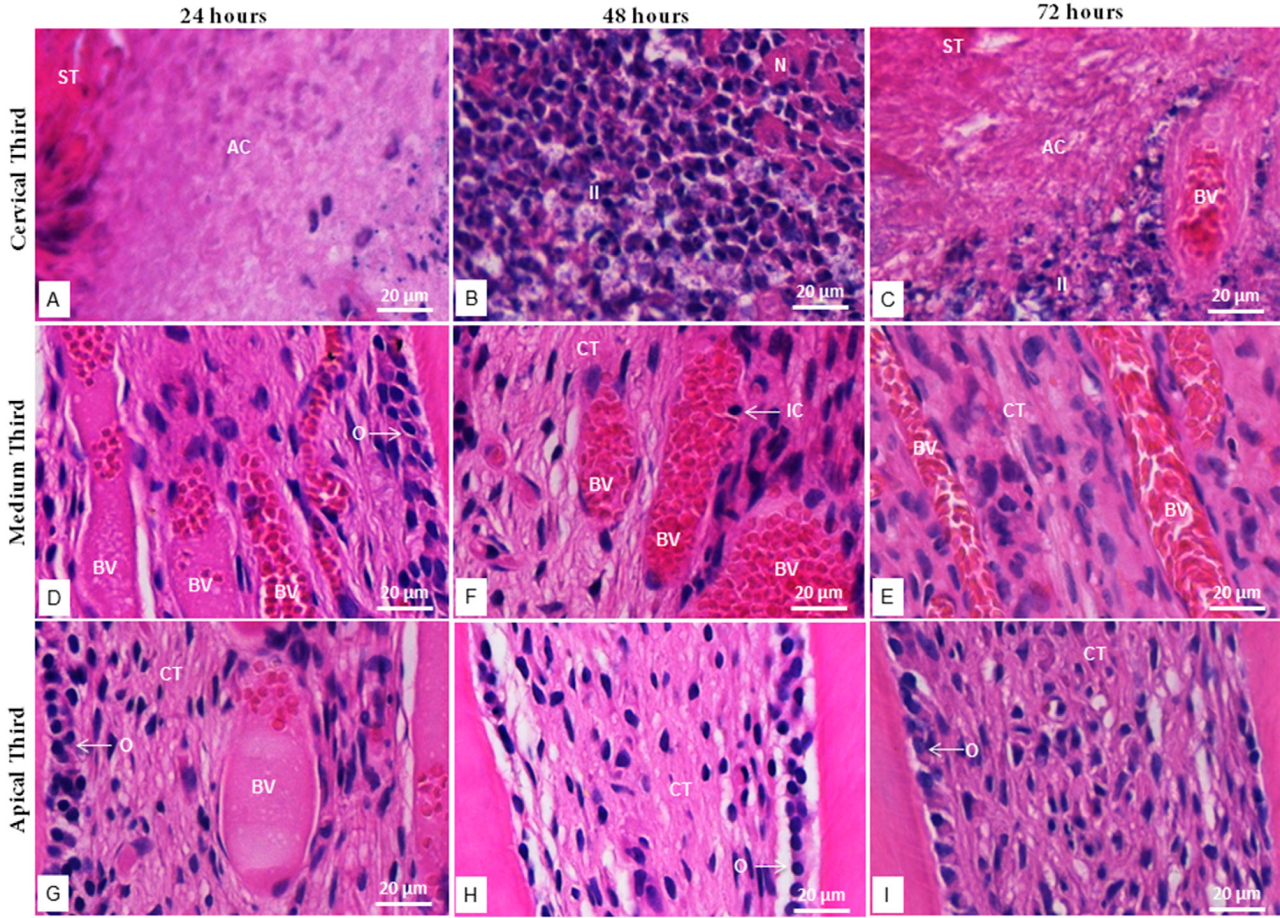
Photomicrographs of hematoxylin and eosin-stained radicular third sections of FS-treated teethat 24, 48 and 72 hours. a-c: Cervical radicular third at 24, 48 and 72 hours, respectively; d-f: Medium radicular third at 24, 48 and 72 hours, respectively; g-i: Apical radicular third at 24, 48 and 72 hours, respectively. (ST) superficial tissue; (AC) acellular layer; (II) inflammatory infiltrate; (N) necrosis; (BV) blood vessels; (CT) connective tissue; (O) odontoblast; (IC) inflammatory cell. 40x magnification

In the MTA group, inflammatory cells were grouped into the tissue surrounding the material, with superficial necrosis in some histological sections, while the deeper tissue was well organized and exhibited several fibroblasts among the bands of collagen fibers, which are features of normality (Figure 3). In the FS group, the tissue adjacent to the material was acellular, followed by a layer of disperse inflammatory cells, sometimes presenting necrotic areas. In some sections, the deeper tissue was scarcely organized (Figure 4).

There was no significant difference in the number of inflammatory cells between MTA and FS groups at 24 (p=0.6410), 48 (p=0.5009) and 72 (p=0.8257) hours by t test. Regarding the period, in the MTA group, the greatest number of inflammatory cells was observed at 48 hours and the lowest at 24 hours (Kruskal-Wallis test p<0.05). There was no difference regarding the 72-hour period in comparison with the others (Kruskal-Wallis test, p>0.05). In the FS group, there was no significant difference in the number of cells between periods (Kruskal-Wallis test, p=0.0551, Table 1). In both groups, a difference was found in the number of inflammatory cells in the radicular thirds (t test, p<0.05), with the greatest amount observed at the cervical third, progressively decreasing through the medium and apical thirds (Table 2).

**Table 1:** Frequency distribution of the means and percentages of inflammatory cells and vascular density in the radicular thirds of remaining pulp tissue after pulpotomy with white MTA or 15.5% FS

Material	Radicular third	Inflammatory cells n (%)	Vascular density n (%)
MTA	Cervical	2992.17 (81.52)^a^	1134.17 (26.94)^a^
	Medium	450.00 (12.94)^b^	1856.50 (44.10)^b^
	Apical	203.17 (5.54)^c^	1219.33 (28.96)^a^
FS	Cervical	3284.17 (77.08)^a^	1105.00 (24.95)^a^
	Medium	737.33 (17.31)^b^	2121.00 (47.89)^b^
	Apical	239.17 (5.61)^c^	1202.83 (27.16)^a^

*Results of Kruskal-Wallis test. Different lowercase letters differ statistically in the same column (p<0.05)

**Table 2:** Frequency distribution of the means and percentages of inflammatory cells and vascular density in the pulp tissue after pulpotomy with white MTA or 15.5% FS over the experimental periods

Material	Periods (h)	Inflammatory cells n(%)	Vascular density n(%)
MTA	24	483.00 (13.16)^a^	1068.00 (25.36)^a^
	48	1745.67 (47.56)^b^	1422.50 (33.78)^a^
	72	1441.67 (39.27)^ab^	1719.50 (40.84)^a^
FS	24	487.00 (11.43)^a^	1360.33 (31.00)^a^
	48	2194.00 (51.49)^a^	1751.67 (39.55)^a^
	72	1579.67 (37.07)^a^	1316.83 (29.73)^a^

*Results of Kruskal-Wallis test. Different lowercase letters differ statistically in the same column (p<0.05)

Vascular density was similar in teeth treated with MTA and FS at 24 (p=0.5320), 48 (p=0.2153) and 72 (p=0.2302) hours, by t test. No significant difference was observed between periods for MTA (p=0.3911) and FS (p=0.3098) groups (Kruskal-Wallis test, Table 1). Greater vascularization was detected in the medium radicular third in teeth, treated with either MTA or FS (Kruskal-Wallis test, p<0.05), with similar vascular density in the cervical and apical thirds (Kruskal-Wallis test, p>0.05, Table 2).

There was no significant correlation between the number of inflammatory cells and the vascular density for both materials (Spearman correlation test, p=0.7189).

**Figure 5:**
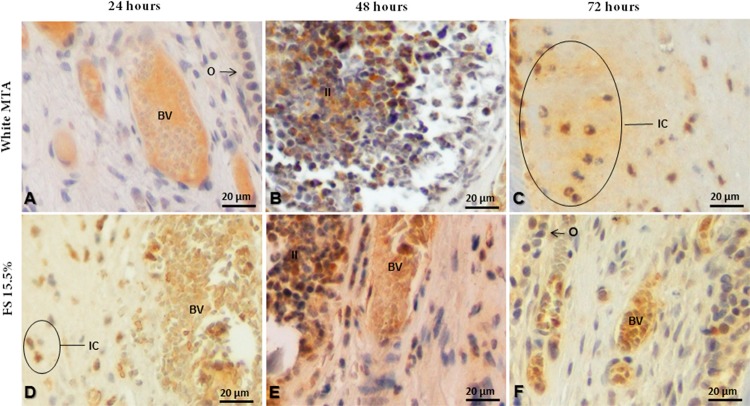
Photomicrographs of IL-6 immunostained cervical or medium radicular third sections of teeth treated with MTA and FS. a-c: MTA group at 24, 48 and 72 hours, respectively; d-f: FS group at 24, 48 and 72 hours, respectively. (IC) inflammatory cell; (O) odontoblast; (II) inflammatory infiltrate; (BV) blood vessels. 40x magnification

Cytoplasmatic expression of IL-6 was observed in odontoblasts and in some inflammatory cells present in pulp tissue after pulpotomy with MTA and FS. Occasionally, a diffuse immunostaining was found in the extracellular matrix of the connective tissue (Figure 5). In the control teeth (sound and untreated ones), no immunostaining was detected.

In the quantification of IL-6 immunostaining, a triple significant interaction between the studied factors was detected in the analysis of variance (F test, p=0.0039); thus, interactions were decomposed. One should consider that high values represent the low IL-6 expression, whereas low values represent the high IL-6 expression.

When comparing the immunostaining caused by either MTA or FS in the same radicular third and at the same experimental period, greatest IL-6 expression was found in the MTA group, mainly in the cervical third at 24 hours (p=0.0074), in the medium third at 72 hours (p=0.0250), and in the apical third at 72 hours (p=0.0331) ─ assessed by Tukey test. Higher immunostaining was found in the apical third at 24 hours, particularly in teeth from the FS group in comparison with the MTA group (Tukey test, p=0.0091, Table 3).

**Table 3:** Mean expression of IL-6 in the radicular thirds of remaining pulp tissue after pulpotomy with white MTA or 15.5% FS over the experimental period

Material	Radicular third	Period (h)	IL-6 expression	Deviation standard
MTA	Cervical	24	120.00^a,A,Ω^	9.3
		48	150.00^b,A,Ω^	14.3
		72	142.25^b,A,Ω^	5.56
	Medium	24	142.25^a,B,Ω^	8.65
		48	154.25^a,A,Ω^	9.25
		72	143.25^a,A,§^	13.81
	Apical	24	170.25^a,C,Ω^	9.14
		48	162.00^a,b,A,Ω^	6.37
		72	150.75^b,A,§^	10.5
FS	Cervical	24	137.50^a,A,§^	10.4
		48	152.25^a,A,Ω^	4.5
		72	142.50^a,A,Ω^	6.95
	Medium	24	145.00^a,A,Ω^	4,00
		48	164.50^b.A.B.Ω^	7.32
		72	157.75^a,b,B,Ω^	4.42
	Apical	24	153.25^a,A,§^	9.63
		48	171.75^b,B,Ω^	10.17
		72	164.50^a,b,B,Ω^	7.32

*Results of Tukey test. Means followed by different lowercase letters indicate statistical differences between the periods of evaluation for the same radicular third of teeth treated with MTA or FS (p<0.05).**Means followed by different capital letters indicate statistical differences between the radicular thirds for the same period of evaluation of teeth treated with MTA or FS (p<0.05). ***Different symbols indicate statistical differences between MTA and FS, for the same radicular third and period of evaluation (p<0.05)

In the comparison between different periods for the same material and the same radicular third, by Tukey test, a statistically significant difference was found in the cervical third of teeth treated with MTA between 24 and 48 hours (p<0.0001), with the highest immunostaining at 24 hours. No difference was found in the cervical third of teeth from the FS group over the periods (p=0.0669). In the medium third of teeth from the MTA group, there was no difference in IL-6 expression over time (p=0.1161). However, for the FS group in the medium third, there was a difference between 24 and 48 hours, with the highest immunostaining at 24 hours (p=0.0105). In the apical third, for the MTA group, there was greater IL-6 expression at 72 hours when compared with 24 hours (p=0.0116), while for the FS group there was greater IL-6 expression at 24 hours in comparison to 48 hours (p=0.0170, Table 3).

The comparison between different radicular thirds for the same material and period revealed a difference between the three thirds for the MTA group at 24 hours, with the highest immunostaining in the cervical third, gradually decreasing through the medium and apical thirds, by Tukey test (p<0.0001). For the FS group at 24 hours, no difference was found regarding IL-6 expression in the radicular thirds (F test, p=0.0514). At 48 hours, no difference was found between the thirds for the MTA group (F test, p=0.1637); however, greater expression was found in the cervical third when compared with the apical third for the FS group (Tukey test, p=0.0109). At 72 hours, there was no difference between the radicular thirds in the MTA group (F test, p=0.3431); however, for the FS group a greater expression was found in the cervical third when compared with the medium and apical thirds (Tukey test, p=0.0031) (Table 3).

## Discussion

The initial inflammatory reaction after pulpotomy is not completely elucidated, and it is believed that pulpotomy failures may be related to undiagnosed, subclinical inflamed pulp.^20^ Therefore, it is important to conduct analyses that seek to understand the pulp repair mechanism after the application of a capping material since an exacerbated initial inflammatory response may cause deeper tissue necrosis.^17^ Considering that inflammation is an early event, we studied the pulp response 24, 48 and 72 hours after the execution of the pulpotomies.

We chose the animal model of the rat mandibular molar free of caries^4,18,21,22^due to ethical aspects, as one cannot extract a human primary tooth right after pulpotomy to evaluate the initial pulp responses to different materials. According to Dammaschke^23^ (2010), rat molars are appropriate to test capping materials. Although this model is not completely clinically relevant, it provides a way to study the cellular and molecular mechanisms that occur during dental pulp repair.

MTA was chosen for this study since it is the gold standard capping material for pulpotomy of primary teeth, due to its high clinical, radiographic and histological success rates.^3,24,25^ However, important limitations of MTA include: tooth discoloration, high cost, considering the survival time of the primary teeth in the oral cavity^5^ and difficulty with handling and insertion due to its grainy consistency and the possibility of it breaking down due to a long setting time and prolonged maturation phase.^26^

The FS is a low-cost alternative to MTA and, according to a systematic review, the clinical success rates after pulpotomy with FS ranges from 78 to 100%, while the radiographic ones stem from 42 to 97%.^27^ Although clinical and radiographic evaluation of pulpotomized teeth using FS has been reported in several studies^1,12,13,15^there are few studies involving histological analysis of the remaining pulp after FS pulpotomies.^4,14,15,28^ The histological assessment of pulp response provides a greater comprehension of its action mechanism.^28^

In the MTA group, a greater number of inflammatory cells was found in the cervical third. Neighboring the material, inflammatory infiltrate and some necrotic areas were observed. Salako, et al.^4^ (2003) also found inflammatory cells surrounding MTA, which was used as capping agent in rat molars, 2 weeks after the treatment. The presence of necrotic tissue suggests that MTA, similarly to calcium hydroxide, initially causes a coagulation necrosis when in contact with the pulp connective tissue due to its high alkalinity.^29^ The alkaline pH and the release of calcium ions evoke the inflammatory reaction. However, calcium ions react with carbon dioxide present in the tissue and originate calcite crystals, thus reducing the inflammatory process. Therefore, the use of MTA as a capping material allows the remaining pulp to remain vital and induces the formation of mineralized tissues, while presenting no deleterious effect to the germ of successor permanent tooth.^24^

In the FS group, the greatest number of inflammatory cells was also found in the cervical radicular third. The tissue in contact with the capping material was reddish in color, followed by an apparently acellular layer. We suggest this morphological feature is due to the hemostatic activity of FS, in which a ferric ion-protein complex is precipitated, thus mechanically blocking the capillary orifices at the surface of pulp stumps.^1,30^ Salako, et al.^4^ (2003) found a moderate inflammation of pulp tissue treated with FS, with a necrotic area localized coronally after 2 and 4 weeks. Koyuturk, et al.^31^ (2013) evaluated the use of Ankaferd blood stopper (ABS) as a pulpotomy agent in rat molars and compared it with FS and formocresol (FC) histologically, observing a higher density of inflammatory cells with the use of FC.

It should be mentioned that the use of a zinc oxide and eugenol (ZOE) as a base was previously considered as responsible for the pulp inflammation after pulpotomies with FS^32^ since eugenol molecules are small and may easily diffuse through pulp tissue, acting as a permanent irritant and causing a subclinical chronic inflammation.^3^ On the other hand, the metal-protein complex formed by FS in contact with blood may behave as a blockade to the irritant components of the ZOE base.^30^ Therefore, this barrier may have restricted the inflammatory process and tissue disorganization caused by ZOE and not by FS.

There were no significant differences in the pulp reaction caused by the studied materials. However, the histological analysis revealed the FS group presented greater pulp disorganization and vascularization in the deeper tissue. Additionally, the inflammatory infiltrate decreased by the third day, indicating the beginning of the inflammatory process resolution.

Reyes-Carmona, et al.^33^ (2010) also observed a decrease in the number of inflammatory cells in the third day of evaluation after pulpotomy with MTA. In that study, neutrophils were the predominant cell-type in the remaining pulp tissue close to the capping material in day 1. Then, neutrophil recruitment diminished from day 1 to day 3, when the migration of macrophages and lymphocytes to the tissue occurred. These findings corroborate those of Cavalcanti, et al.^34^ (2011), who reported the early immune cells recruited to the wound pulp are neutrophils, which play a key role in cytokine release and phagocytosis.

In a clinical study histologically assessing teeth treated with FS and MTA 15 months after pulpotomy, Junqueira, et al.^15^ (2018) found that the remaining radicular pulp from both groups were vital and without inflammatory cell infiltration. Another study compared the histological alterations in the pulp tissue 30 and 40 days after pulpotomy with FS and laser; the authors verified that the ability of both materials to induce a foreign body reaction and an inflammatory cell response in the pulp tissue was not significant in both groups.^28^

Considering that pulpotomy agents have been considered as a factor affecting the outcome of vital pulp treatment, research has been performed to evaluate the relationship between cytokines and capping materials such as CH, MTA and FC.^34^ It is suggested that IL-6 is a suitable indicator of pulp condition and its monitoring may enhance the accuracy of prognosis of vital pulp therapy.^35^

Studies employing the rat subcutaneous implantation model showed an association between the reduction of inflammatory process and the down-regulation of IL-6 in capsules surrounding the implanted calcium silicate-based cements.^36,37^ However, no study has evaluated the expression of IL-6 in rat pulps after the use of MTA or FS.

Since in this study a weak to moderate IL-6 immunostaining was observed after the pulp treatment with MTA and FS, it is suggested the occurrence of a mild pulp inflammation. In addition, a greater IL-6 expression was observed in the cervical radicular third of teeth from both groups over the experimental period, suggesting its participation in the inflammation caused by capping agents during pulp healing. MTA may inhibit or induce the secretion of cytokines such as IL-6 by cells from the connective tissue around the material.^36^ This material promotes leukocyte recruitment from the blood vessels, initially inducing an inflammatory process in the adjacent tissues and stimulating the secretion of inflammation-modulating cytokines.^38^

The pulp response to a material is the best criterion to judge the performance of a capping agent used for pulpotomy.^39^ Histological and immunohistochemical analyses have proven that clinical and radiographic successes are not consistent with the true state of pulp health.^40^ Therefore, although the periods of observation are relatively short, this study brought new insight into the initial molecular and cellular events that might lead to pulp repair after pulpotomy with MTA and FS.

## Conclusion

The radicular pulp of MTA-treated teeth often presented better histological features and greater IL-6 expression than those treated with FS. However, there was no difference between the groups regarding the inflammatory status and vascularization, suggesting the usefulness of FS as a low-cost alternative to MTA. Further studies should be conducted to confirm the histological success of FS after pulpotomy of primary teeth.
